# Intraspecific variation of the hedgehog arteriviruses, which may constitute a new genus in the subfamily *Heroarterivirinae* of the family *Arteriviridae*

**DOI:** 10.1007/s00705-025-06231-7

**Published:** 2025-02-08

**Authors:** Akbar Dastjerdi, Hannah Davies, Nadia Inglese, Samantha Holland, Dmitry V. Samborskiy, Alexander E. Gorbalenya

**Affiliations:** 1 Animal and Plant Health Agency (APHA)-Weybridge, Addlestone, Surrey KT15 3NB UK; 2 Belozersky Institute of Physico-Chemical Biology, Lomonosov Moscow State University, Moscow, 119899 Russia; 3 Leiden University Center of Infectious Diseases, Leiden University Medical Center, P.O. Box 9600, E4-P, 2300 RC Leiden, The Netherlands

## Abstract

We recently discovered a novel member of the family *Arteriviridae*, hedgehog arterivirus 1 (HhAV-1), in the brains of hedgehogs with fatal encephalitis. In this study, we classified this virus and investigated its intrahost genomic diversity using next-generation sequencing. We sequenced HhAV-1 genomes from specimens from seven hedgehogs (two males and five females) with signs of encephalitis that were collected in Buckinghamshire, Gloucestershire, and Cambridgeshire, England, and had died or been euthanised between 2013 and 2024. Analysis of the intrahost populations of these seven HhAV-1 isolates and a previously described isolate revealed the presence of single-nucleotide polymorphisms (SNPs), which were most frequent in open reading frames 5, 6, and 7, encoding glycoprotein 5, the membrane protein, and the nucleocapsid protein. Pairwise comparisons of the eight HhAV-1 variants showed that the nucleotide sequence identity values in their combined complete coding sequences ranged from 76.2% to 100%. The eight HhAV-1 variants also shared at least 82.8% amino acid sequence identity in five domains that are involved in replication and are used for the classification of nidoviruses: 3CLpro, NiRAN, RdRp, ZBD, and HEL1. In a replicase-based phylogenetic tree of members of the family *Arteriviridae*, the HhAV-1 variants formed a sister cluster to African pouched rat arterivirus. A DEmARC-based pairwise distance analysis indicated that these viruses may comprise a new species, for which we propose the name "*Xiarterivirus erinaceid*", in a new genus in the subfamily *Heroarterivirinae*.

**Supplementary Information:**

The online version contains supplementary material available at 10.1007/s00705-025-06231-7.

Arteriviruses are enveloped spherical viruses with a positive-sense, single-stranded linear RNA genome with short 5’ and 3’ untranslated (UTRs) that flank two functionally different protein-encoding genomic regions [27]. The 5′ protein region contains two overlapping open reading frames (ORFs), 1a and 1b, coding for 12 non-structural proteins (nsp1-nsp12) that form a replicative-transcription complex (RTC). These non-structural proteins include the five most conserved and essential domains: the ORF1a-encoded main protease (M^pro^) domain, also known as 3CL^pro^, in nsp5, the ORF1b-encoded nidovirus RdRp-associated nucleotidyltransferase (NiRAN) and RNA-dependent RNA polymerase (RdRp) domains in nsp9, and the cysteine-rich zinc-binding domain (ZBD) and nucleoside triphosphate-binding/helicase (HEL1) domain in nsp10 [13]. The 3′ protein-encoding region encodes the structural components of the virion, with at least seven partially overlapping ORFs: ORF2-ORF7 [27]. These ORFs code for the envelope protein (E), the glycoproteins GP2-5, the membrane (M) protein, and the nucleocapsid (N) protein. The conservation of the five replicative protein domains, 3CLpro (located in nsp4), NiRAN and RdRp (nsp8-9), and ZBD and HEL1 (nsp10) has enabled the establishment of a common framework for the taxonomy of the family *Arteriviridae* and other families of the order *Nidovirales*. Accordingly, arteriviruses are assigned to six subfamilies: *Crocarterivirinae*, *Equarterivirinae*, *Heroarterivirinae*, *Simarterivirinae*, *Variarterivirinae*, and *Zealarterivirinae*, with 13 genera and 23 species [3].

Arteriviruses are highly host-species-specific and establish a prolonged infection in their natural hosts [1]. The epidemiology and pathogenesis of the infections caused by each arterivirus is distinct, as is the associated disease. The initial discovery of arteriviruses dates to the early 1950s, with the description of equine arteritis virus (EAV) in 1953 [4], and porcine reproductive and respiratory syndrome virus (PRRSV; renamed as PRRSV-1 to distinguish it from the subsequently identified PRRSV-2) was described in the late 1980s [7, 25, 32]. The arteriviruses discovered in 2012 include wobbly possum disease virus (WPDV) [10] and four genetically divergent simian haemorrhagic encephalitis viruses (SHFVs) representing different virus species [19, 31]. New arteriviruses were also described in Chinese softshell turtles (*Pelodiscus sinensis*) with haemorrhagic disease in 2013 [23], healthy African giant shrews (*Crocidura olivieri*) in 2016 [30], and most recently, in the brains of European hedgehogs (*Erinaceus europaeus*) with encephalitis (hedgehog arterivirus 1, HhAV-1) in 2019 by our group [9].

Hedgehogs are solitary nocturnal animals that stay in the same location during their lifetime. These characteristics limit their contacts and opportunities to transmit microbial pathogens in the wild. However, an ongoing loss of their natural habitat in rural areas of the United Kingdom (UK) is progressively causing hedgehogs to be displaced into parks and gardens of homes, increasing their direct contacts and the potential for transmission of pathogens. In addition, the loss of habitat subjects hedgehogs to frequent road accident injuries and hospitalisation, increasing the incidence of close exposure to other hedgehogs or other animal species in care facilities. It can therefore be predicted that outbreaks of infectious diseases of hedgehogs, such as those caused by HhAV-1, and the evolution and potential transmission of these viruses to other animal species will increase over time.

Molecular characterisation of arteriviruses has been of significant research interest, considering their genetic diversity, ecology, and economic impact. For example, two of the most economically important arteriviruses, PRRSV-1 and PRRSV-2, are further divided into several subtypes, each apparently circulating in separate geographic areas [26, 28]. Furthermore, sequence analysis of PRRSV-1 isolates worldwide has shown striking genetic diversity (mainly in nsp1, nsp2, ORF 3 [GP2], and ORF4 [GP3]) due to substitution and recombination [5, 8, 24]. This variability also affects most of the known linear B-cell epitopes, resulting in considerable antigenic diversity of PRRSV-1 and PRRSV-2. Analysis of these viruses within a taxonomic framework has revealed that these two serotypes diverged profoundly, even in the most conserved replicase proteins, and accordingly comprise two different arterivirus species [3]. This indicates that rigorous distance-based analysis, in addition to consideration of host specificity and pathology, may be required for proper classification of arteriviruses.

In this study, we identified and genetically characterised HhAV-1 in hedgehogs displaying clinical signs of encephalitis and analysed the intra-host population genetic variation of this virus to clarify its taxonomic classification. We also addressed the potential impact of the genetic diversity of HhAV-1 on hedgehog populations.

Between 2013 and 2024, seven hedgehogs (two males and five females) were admitted to a hospital and care centres for disease, minor injuries, malnutrition, parasite burden, and orphaning, and they became sick on site (Supplementary Table S1). Clinical signs in most sick animals were consistent with those of encephalitis, and all of the hedgehogs died or were euthanised.

Post-mortem brain tissues (15–20 mg) were homogenised using a gentleMACS homogeniser, and RNA was extracted using 1 ml of TRIzol LS Reagent (Fisher Scientific) in a gentleMACS M tube (Miltenyi Biotec). The homogenate was centrifuged at 2000 × *g* for 3 min to remove large pieces. The supernatant (200 μl) was transferred to an Eppendorf tube containing 200 μl of chloroform, vortexed vigorously for 15 s, left at room temperature for 2–3 minutes, and centrifuged for 10 min at 12,000 × *g* at 2 to 8°C. The upper aqueous phase (140 μl) was transferred to an Eppendorf tube containing 560 μl of AVL buffer (QIAGEN). Viral RNA was extracted using a QIAamp Viral RNA Mini Kit (QIAGEN) according to the manufacturer’s instructions. A real-time reverse transcription quantitative polymerase chain reaction (RT-qPCR) assay for HhAV-1 was performed as described by Dastjerdi et al. [9].

Next-generation sequencing (NGS) was performed either on-site at the Animal and Plant Health Agency (APHA) Weybridge using an Illumina NextSeq platform or at Source BioScience (Cambridge, UK) on a NovaSeq X Plus platform. The arterivirus sequences were assembled *de novo* or mapped to the published sequence of HhAV-1 strain 0073 (GenBank accession number MT415062.2) using SeqMan NGen 17.5 software (DNASTAR, Inc.). Analysis of single-nucleotide polymorphisms (SNPs) and minor variants was carried out for each specimen, using the "Variant analysis/Resequencing" option of the SeqMan NGen 17.5 software. Raw sequence reads were mapped to the corresponding annotated sequence for each virus with a minimum percentage of variation of >5%. The "Variant analysis/Resequencing" option of SeqMan NGen was used for the analysis with default settings and the "somatic/cancer/heterogenous" and "unknown sex" options. The analysis was performed using complete coding sequences (CDSs) of the genome unless otherwise stated. Also, the raw data for the published sequence MT415062.1 were re-analysed to update the genome sequence and identify SNPs. Since all of the genome sequences of arteriviruses used in this study and that of MT415062.2 were found to be closely related and formed a species-like cluster (see below), we will refer to these viruses as isolates of HhAV-1. To distinguish these isolates, their names include isolate-specific suffixes (Supplementary Table S1).

Multiple sequence alignments of the five most conserved replicative protein domains (3CLpro, NiRAN, RdRp, ZBD, and HEL1) of eight HhAV-1 variants with those of other arteriviruses was carried out using MegAlign 15 software with an arterivirus-wide alignment template provided by the *Arteriviridae* Study Group (https://ictv.global/report/chapter/arteriviridae/arteriviridae/resources). This alignment was used to produce a phylogenetic tree for 23 arteriviruses, representing all of the species in the family [3], and eight HhAV-1 isolates by the maximum-likelihood method with the Le_Gascuel_2008 model [20] and 500 bootstrap replicates [Felsenstein, 1985], using the MEGA X software package [16]. Provisional classification of HhAV-1 within the *Arteriviridae* taxonomy framework was done using the same protein alignment for these species but including 1500 sequences representing the current sampling of different species using the Viralis software platform [12]. The analysis was performed with the assistance of the DEmARC software for cost-based analysis of pairwise evolutionary distances (PED) [17, 18] and by using established rank thresholds for the family [3].

Post-mortem brain specimens of the seven hedgehogs studied here had a relatively high load of HhAV-1, with quantification cycle (Cq) values in a range of 19 to 28.9 (Supplementary Table S1), in apparent association with their clinical signs. The high viral load allowed recovery of nearly complete viral genome sequences from the NGS raw sequence data (reads), whose median coverage per genome ranged from 24.87 to 260.33 reads for the eight isolates (Supplementary Table S2). The genome sequences of the seven new HhAV-1 isolates have been deposited in the NCBI GenBank database under accession numbers PP432684 to PP432688, PP885722, and PP885723. The genome sequence of the prototype HhAV-1 strain 0073 (MT415062.1) was updated at 16 nucleotide positions and is now available under accession number MT415062.2.

We re-examined the NGS data for each of the eight HhAV-1s – the seven from this study and another published previously by us [9] – for SNPs or minor variants relative to the annotated consensus sequence of the respective HhAV-1 strain (Supplementary Table S3). For the combined CDSs of the eight genome sequences, between 24 and 198 SNPs were identified in different isolates with a frequency threshold of >5%, with the fraction of reads with variations within the CDSs reaching up to 47.8% (Supplementary Table S3A). The depth of the sequence reads, the number of reads that mapped to the SNP (or a short region in the case of an indel), varied from 3 to 830 reads for the entire virus dataset and also varied substantially for separate isolates. On average, ORF5, 6, and 7 were found to have the highest percentage of SNPs, while ORF1a, 1b, 2a, 2b, 3, and 4 had the lowest. The average number of SNPs per virus CDS set in this dataset was 29.9 synonymous SNPs, 51.3 non-synonymous SNPs, and 5.5 SNPs that could result in a frameshift, nonstop, nonsense, or in-frame insertion or deletion (Supplementary Table S1B). No SNPs of the last category were identified in ORF2b, but they were observed with the highest frequency in ORF7, which also had the most synonymous and non-synonymous SNPs.

The pairwise nucleotide sequence identity for the combined CDSs of the eight HhAV-1 isolates (ranging in length from 13,452 to 13,584 nucleotides) in all 28 virus pairs ranged from 76.2% (0045 and 0047 vs. 1123) to 99.9% (0045 vs. 0047) (Supplementary Table S2). When this pairwise analysis was extended to proteins of seven HhAV-1 strains (excluding 0045, as it was identical to 0047), GP3 and the other three glycoproteins, GP2, GP4, and GP5, were found to be the most divergent, with median pairwise identity varying from 68% to 80% (Fig. 1). In contrast, the four most conserved proteins, nsp5, nsp8-9, nsp10, and N, had pairwise median identity values ranging from 92% to 93%. The pairwise variation for the other 10 viral proteins ranged from 82% to 91%, with nsp2 being the most divergent among the non-structural proteins (82%).

**Fig. 1:**
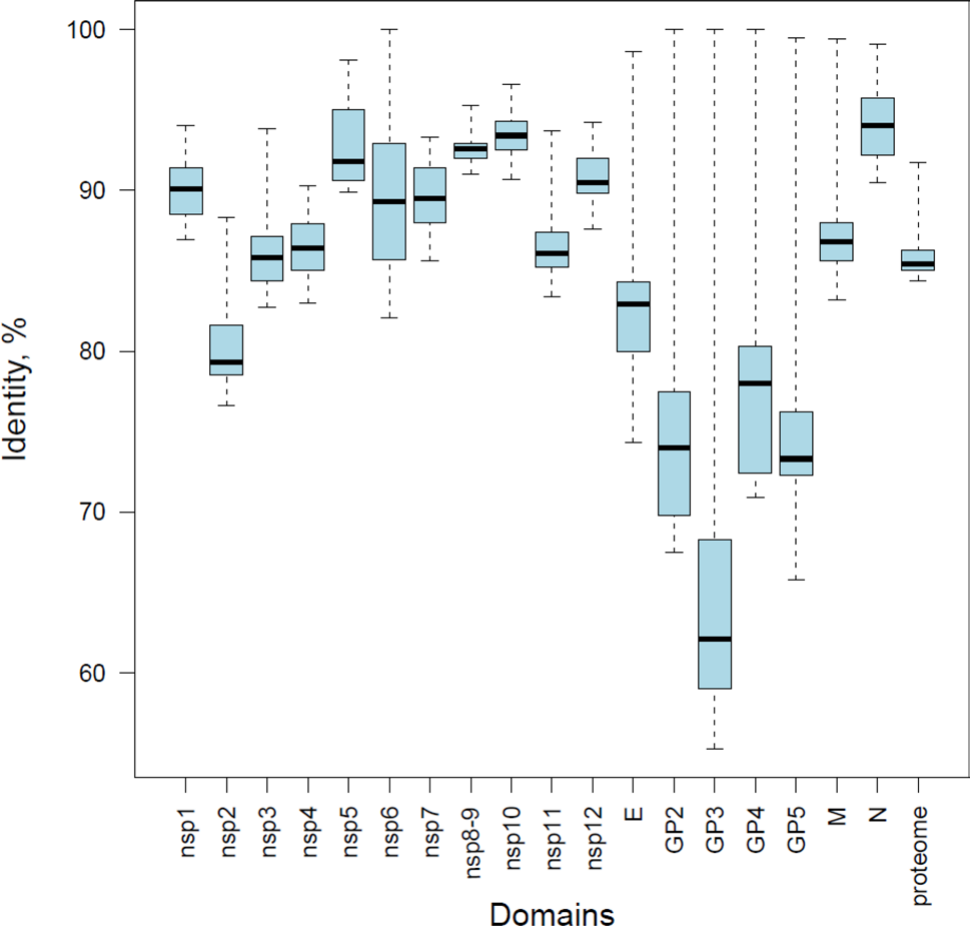
Amino acid sequence divergence of the proteins of HhAV-1 isolates. Shown is a box-and-whisker plot of the pairwise sequence identity of the proteins of seven HhAV-1 isolates (Supplementary Table S1). Isolate 0045 was excluded from this analysis because its protein sequences were found to be identical to those of isolate 0047. The entire and 25-to-75% distance ranges and median distance are depicted for nsp1-12 and the structural proteins. For this analysis, overlapping parts of structural proteins were used only for the most distal protein in a pair, e.g., for GP5 in the GP4 and GP5 pair.

In line with the above results (Fig. 1), the eight HhAV-1 variants shared at least 83.0% amino acid sequence identity in the five replicative domains of nsp4, nsp8-9, and nsp10 (3CLpro, NiRAN, RdRp, ZBD, and HEL1) that are used for the classification of nidoviruses [13]. In contrast, they shared only 33.4–55.5% amino acid sequence identity with the 23 arteriviruses representing all of the species in the family. The highest pairwise identity (55.5%) was with African pouched rat arterivirus (APRAV), the prototype of the genus *Lambdaarterivirus* in the subfamily *Heroarterivirinae*. Excluding this virus, the highest amino acid sequence identity for these five domains between HhAV-1 and the members of the other 22 arterivirus species was 48.6%. These data indicate that the hedgehog arteriviruses are distinct from previously described arteriviruses.

Phylogenetic analysis of the HhAV variants and representatives of 23 arterivirus species showed that the HhAVs formed a distant sister branch to APRAV (Fig. 2). The DEmARC-based analysis of inter-virus PEDs revealed the intragroup variation of the HhAVs to be below the PED threshold, with the HhAVs and APRAV being separated by a PED range that exceeds the genus PED threshold but is below the PED subfamily threshold for the family *Arteriviridae* (Fig. 3). These results imply that the HhAV-1 variants may represent a new species and a new genus in the subfamily *Heroarterivirinae*. For this new species, we propose the name "*Xiarterivirus erinaceid*", using a binomial nomenclature including a genus-specific stem ("*Xiarterivirus*"; using the letter Xi of the Greek alphabet as a prefix) and a species-specific suffix ("*erinaceid*"; reference to hedgehogs in Latin). These names conform to the nomenclature format adopted by the *Arteriviridae* Study Group of the International Committee on Taxonomy of Viruses (ICTV) [3] but will require ICTV approval. DEmARC pairwise distance analysis revealed the intraspecific variation of HhAV-1 to be comparable to those of the most variable species, including two PRRSVs, EAV, Free State vervet virus (FSVV), Mikumi yellow baboon virus 1 (MYBV-1), Kibale red-tailed guenon viruses 1 (KRTGV-1), and WPDV (Fig. 3).

**Fig. 2:**
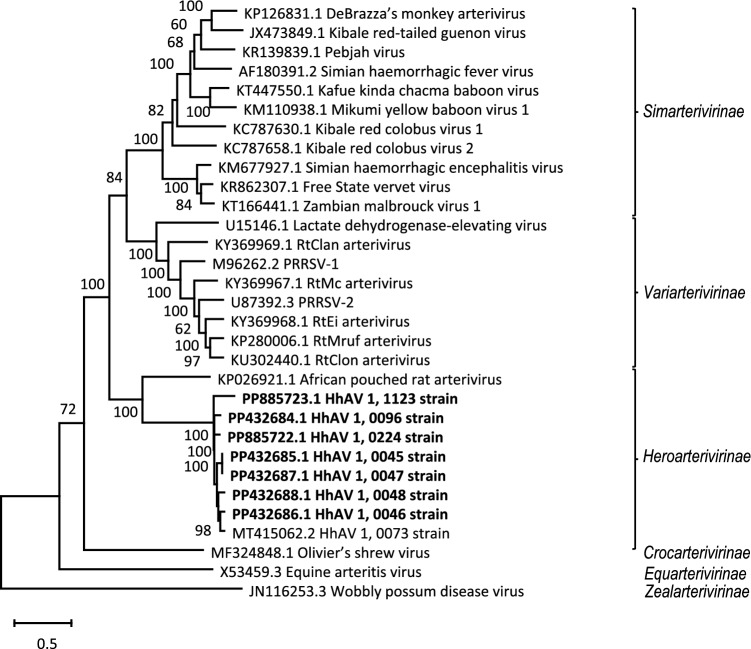
Phylogenetic analysis of arteriviruses. For virus selection, alignment, and tree generation using MEGA X software, see Materials and methods. The tree with the highest log likelihood (−32651.18) is shown. The percentage of trees (bootstrap values) in which two or more viruses clustered together is shown next to the respective branches. The tree is drawn to scale, with branch lengths measured in the number of substitutions per site. Bootstrap values less than 50% have been omitted. Arterivirus subfamilies are shown at the right. Each virus on the tree is represented by its GenBank accession number and name. The designation of subfamilies is according to Brinton et al. [3]. The viruses analysed in this study are shown in bold.

**Fig. 3:**
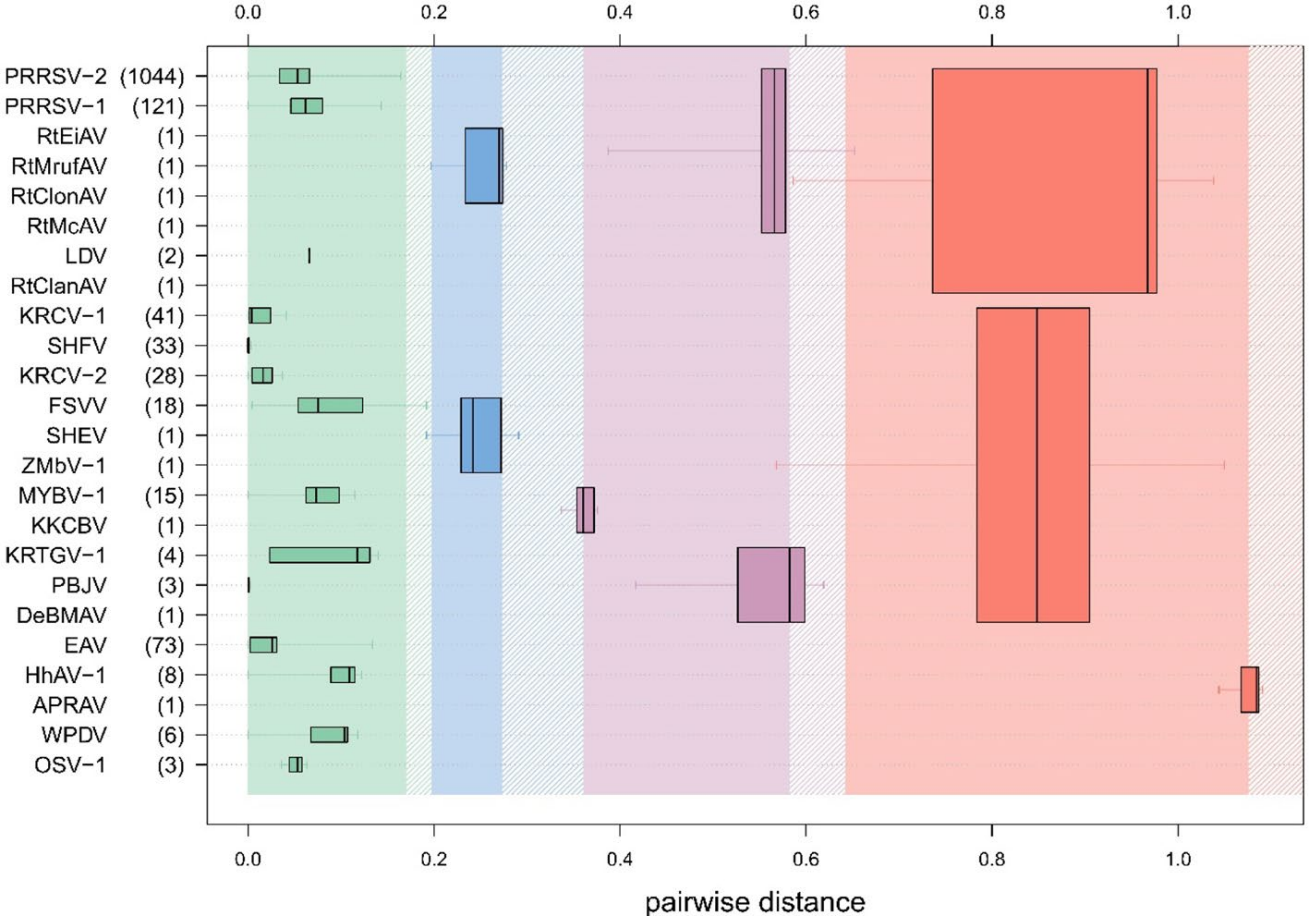
Hierarchical classification of arteriviruses according to DEmARC. Presented is the distribution of pairwise distances using a box-and-whiskers diagram for clusters at four levels that correspond to taxonomy ranks of the family *Arteriviridae*, from species to subgenus to genus to subfamily (from lowest to highest). Each level of a classification is coloured differently and shown from left to right. The number of virus strains/isolates/variants used in the analysis for each virus species is shown in parentheses after the abbreviation of the prototype virus for the respective species. PRRSV-1 and PRRSV-2, porcine reproductive and respiratory syndrome virus 1 and 2, respectively; RtEiAV, RtEi arterivirus; RtMrufAV, RtMruf arterivirus; RtClonAV, RtClon arterivirus; RtMcAV, RtMc arterivirus; LDV, lactate dehydrogenase-elevating virus; RtClanAV, RtClan arterivirus; KRCV, Kibale red colobus virus; SHFV, simian haemorrhagic fever virus; FSVV, Free State vervet virus; SHEV, simian haemorrhagic encephalitis virus; ZMbV, Zambian malbrouck virus; MYBV, Mikumi yellow baboon virus; KKCBV, Kafue kinda chacma baboon virus; KRTGV, Kibale red-tailed guenon virus; PBJV, Pebjah virus; DeBMAV, DeBrazza’s monkey arterivirus; EAV, equine arteritis virus; APRAV, African pouched rat arterivirus; WPDV, wobbly possum disease virus; OSV-1, Olivier's shrew virus 1. For other details of the plot design, see Lauber & Gorbalenya [18].

Although our study was limited to the HhAV-1 strains from eight sick animals collected in a geographically restricted area over few years, we already observed considerable intraspecific virus variation comparable to that observed in the three most extensively sampled arteriviruses: PRRSV-1, PRRSV-2, and EAV. In this respect, HhAV-1 resembles a few other poorly studied but diverse arteriviruses isolated from monkeys, e.g., FSVV, KRTGV-1, and MYBV-1 [2] and possums (WPDV) [6]. This intraspecific variation was distributed highly unevenly along the HhAV-1 genome in a functionally sound pattern that conforms to what has been documented for the family *Arteriviridae* [14, 21, 22]. This suggests that the information obtained for other arteriviruses could be applied to HhAV-1, and further characterization of the HhAV-1 infections might provide new insights about the family in general.

The full extent of the natural diversity of the HhAV-1 may prove to be even greater once more clinical samples become available and are sequenced or HhAVs are sequenced from asymptomatic animals. The habitat characteristics of hedgehogs, which are solitary and sedentary animals, and their relatively small population size may limit transmission of the associated viruses and the extent of their intraspecific diversity. On the other hand, hedgehog habitat loss may promote evolution and transmission of HhAV-1, including crossing host species barriers. Indeed, simian haemorrhagic fever virus and other closely related arteriviruses appear to have been transmitted between African primate hosts and Asian macaques on several occasions [15, 29].

Intra-host viral progeny diversity enables a virus to escape the host immune response during infection, to alter its tissue tropism, and to cross host species barriers upon subsequent infection [11, 33]. While the intra-host variation might have been captured in the analysis of SNPs in our study, practically all the SNPs in the sequenced specimens were present at a low frequency and did not affect the respective consensus sequences. We observed that ORF6 and ORF7, which code for the structural membrane protein and nucleocapsid protein, respectively, had the most SNPs in the HhAV-1 strains. However, the SNP analysis presented here must be treated as preliminary, since the NGS coverage depth varied considerably among the different sites and among different HhAV-1 variants and was particularly low for the isolates 0046 and 0047 (Supplementary Table S1). This coverage variation may have contributed to the striking differences in the number and distribution of SNPs in isolates 0047 and 0045 (Supplementary Tables S1 and S3), which otherwise have identical consensus genomic sequences of 13,452 nucleotides (Supplementary Table S2). This is indicative of virus transmission between these two animals, which were kept in the same facility over a short period of time.

The current five-rank classification of arteriviruses assigns a virus to existing or new taxa based on family-wide rank thresholds for pairwise distances in concatenated sequence alignments of the five most conserved domains, which are spread throughout pp1ab: 3CLpro, NiRAN, RdRp, ZBD, and HEL1. Employing this computational framework, we concluded that the newly described genomic diversity of the eight HhAV-1 isolates seems to conform to intraspecific variation and supports classifying this virus as the prototype of a new genus in the subfamily *Heroarterivirinae*, which currently includes a single genus with a single species that was established based on analysis of single genome sequence with no consideration of pathology.

In summary, the HhAV-1 isolates examined in this study should be classified as members of a new species and a new genus in the family *Arteriviridae*. Although preliminary, this study details for the first time the intra-host genetic variation of HhAV-1 during infection and the extent of its natural diversity in hedgehogs in England. Clearly, analysis of more clinical specimens will be required to gain further insights into the genetic evolution of HhAV-1 and to understand its biological effects, epidemiology, and transmission dynamics.

## Supplementary Information

Below is the link to the electronic supplementary material.

Supplementary file1 (DOCX 36 KB)

## Data Availability

The datasets generated in the current study are available in the National Center of Biotechnology Information [NCBI] repository (https://www.ncbi.nlm.nih.gov/) under the accession numbers PP432684 to PP432688, PP885722 and PP885723.

## Abbreviations

ZBD: Zinc-binding domain
RdRp: RNA-dependent RNA polymerase
3CLpro: 3C-like protease
NiRAN: Nucleotidyltransferase domain
HEL1: Superfamily 1 helicase
HhAV-1: Hedgehog arterivirus 1
SNP: Single-nucleotide polymorphism
CDS: Coding DNA sequence
NGS: Next-generation sequencing
PED: Pairwise evolutionary distance
UTR: Untranslated region
nsp: Non-structural protein
RTC: Replicative-transcription complex
E: Envelope protein
GP: Glycoprotein
M: Membrane protein
N: Nucleocapsid protein
