# Supplementary material for: Intraspecific variation of the hedgehog arteriviruses, which may constitute a new genus in the subfamily Heroarterivirinae of the family Arteriviridae
Source: Arch Virol. 2025 Feb 8;170(3):49. doi: 10.1007/s00705-025-06231-7 (PMC11807078; doi:10.1007/s00705-025-06231-7)
Supplement: Supplementary file 1 — Supplementary file1 (DOCX 36 KB) [file 705_2025_6231_MOESM1_ESM.docx]

**Supplementary Table 1:** Hedgehogs positive for HhAV-1 in brain and kidney tissues.

| Hedgehog ID | Age | Sex | Care centre^a^ | Sampling date | Cq value | HhAV-1 strain | GenBank ID |
| --- | --- | --- | --- | --- | --- | --- | --- |
| 1 | Juvenile | F | A | Feb. 2013 | 22.7 | 0096 | PP432684 |
| 2 | Adult | M | B | Dec. 2017 | 19.1 | 0045 | PP432685 |
| 3 | Juvenile | F | B | Feb. 2018 | 22.3 | 0046 | PP432686 |
| 4 | Juvenile | F | B | Jan. 2018 | 19.8 | 0047 | PP432687 |
| 5 | Juvenile | M | B | Jan. 2018 | 20.7 | 0048 | PP432688 |
| 6 | Adult | F | C | Feb. 2024 | 19.6^b^ | 0224 | PP885722 |
| 7 | Adult | F | C | Dec. 2023 | 25.9 | 1123 | PP885723 |
| 8 | Juvenile | M | A | Oct. 2019 | 24.4 | 0073 | MT415062 |

^a^; A, located in Gloucestershire, England; B, located in Buckinghamshire, England; C, located in Huntingdon, Cambridgeshire.

^b^; Kidney tissue, brain tissue had a Ct value of 28.98.

**Supplementary Table 2:**

Detailed information on the NGS data generated for the eight HhAV-1 positive brain samples.

| Animal ID | Total seq. reads | Assembled Seq. reads for HhAV-1 | Contig length | Median coverage | HhAV-1 strain | GenBank ID |
| --- | --- | --- | --- | --- | --- | --- |
| 1 | 39,296,910 | 9,083 | 13,529 | 101.78 | 0096 | PP432684 |
| 2 | 26,759,616 | 4,989 | 13,452 | 77.53 | 0045 | PP432685 |
| 3 | 7,464,288 | 3,321 | 13,506 | 42.16 | 0046 | PP432686 |
| 4 | 3,232,767 | 2,020 | 13,452 | 24.87 | 0047 | PP432687 |
| 5 | 6,265,046 | 4,328 | 13,509 | 56.89 | 0048 | PP432688 |
| 6 | 46,229,706 | 20,231 | 13,487 | 196.66 | 0224 | PP885722 |
| 7 | 66,798,826 | 27,623 | 13,584 | 260.33 | 1123 | PP885723 |
| 8 | 7,291,562 | 4,696 | 13,493 | 64.95 | 0073 | MT415062 |

**Supplementary Table 3: SNP analysis of eight HhAV-1 NGS datasets.**

Minor variants (SNPs) detected in the eight HhAV-1 NGS datasets comparing each virus NGS reads to the corresponding annotated consensus with minimum variant percentage set at > 5%. The Table (A) also contains total number of SNPs, SNP % (the fraction of the reads with the variant call) and depth of coverage (number of reads that mapped to the SNP region) detected in each dataset. Number of SNPs detected in individual ORFs, and their percentages are also shown. Classification of the SNPs in the nine ORFs are reported in Table B. ORF columns contain number of SNPs for each category (synonymous, non-synonymous and frameshift, non-stop, nonsense and Indels) and their percentages.

**A:**

|  |  |  |  | SNP/% | SNP/% | SNP/% | SNP/% | SNP/% | SNP/% | SNP/% | SNP/% | SNP/% |
| --- | --- | --- | --- | --- | --- | --- | --- | --- | --- | --- | --- | --- |
| Strains | Total SNPs | SNP % | Depth | ORF1a | ORF1b | ORF2a | ORF2b | ORF3 | ORF4 | ORF5 | ORF6 | ORF7 |
| 0096 | 115 | 5-47.8 | 24-170 | 54/0.9 | 22/0.5 | 0/0 | 6/0.8 | 11/2 | 11/2.4 | 3/0.5 | 4/0.8 | 4/1.1 |
| 0045 | 52 | 5-16.6 | 12-111 | 41/0.7 | 3/0.1 | 1/0.4 | 1/0.1 | 0/0 | 0/0 | 2/0.3 | 0/0 | 4/1.1 |
| 0046 | 63 | 5-50 | 4-54 | 38/0.6 | 4/0.1 | 2/0.9 | 2/0.3 | 0/0 | 0/0 | 2/0.2 | 8/1.6 | 7/2 |
| 0047 | 198 | 5-33.3 | 3-37 | 121/2 | 13/0.3 | 4/1.7 | 8/1.1 | 1/0.2 | 5/1.1 | 33/5.3 | 13/2.5 | 0/0 |
| 0048 | 73 | 5-20 | 5-78 | 10/0.2 | 16/0.4 | 1/0.4 | 0/0 | 1/0.2 | 0/0 | 4/0.6 | 14/2.7 | 27/7.7 |
| 0224 | 106 | 5-47.8 | 39-649 | 51/0.8 | 21/0.5 | 1/0.4 | 6/0.8 | 9/1.6 | 2/0.4 | 11/1.8 | 3/0.6 | 5/1.4 |
| 1123 | 58 | 5-25 | 8-830 | 45/0.7 | 6/0.1 | 0/0 | 0/0 | 2/0.4 | 1/0.2 | 1/0.2 | 2/0.8 | 1/0.3 |
| 0073^a^ | 24 | 5-11.3 | 16-88 | 8/0.1 | 1/.01 | 0/0 | 0/0 | 1/0.2 | 1/0.2 | 0/0 | 8/1.6 | 5/1.4 |
|  |  |  | Average | 46/0.8 | 10.8/0.2 | 1.1 /0.5 | 2.9/0.4 | 3.1/0.6 | 2.5/0.5 | 7/1.1 | 6.8/1.3 | 6.6/1.9 |

^a^; Accession number MT415062.2 (Dastjerdi et al., 2021)

**B:**

|  | **Strain** | **ORF1a** | **ORF1b** | **ORF2a** | **ORF2b** | **ORF3** | **ORF4** | **ORF5** | **ORF6** | **ORF7** | **Total** |
| --- | --- | --- | --- | --- | --- | --- | --- | --- | --- | --- | --- |
| Synonymous | 0096 | 38^a^/0.6^b^ | 15/0.3 | 0/0 | 1/0.1 | 2/0.4 | 7/1.5 | 0/0 | 3/0.6 | 3/0.9 | 69 |
|  | 0045 | 9/0.1 | 2/0.05 | 0/0 | 0/0 | 0/0 | 0/0 | 0/0 | 0/0 | 2/0.6 | 13 |
|  | 0046 | 11/0.2 | 2/0.05 | 0/0 | 0/0 | 0/0 | 0/0 | 0/0 | 2/0.4 | 2/0.6 | 17 |
|  | 0047 | 23/0.4 | 5/0.1 | 1/0.4 | 4/0.5 | 1/0.2 | 1/0.2 | 7/1.1 | 1/0.2 | 0/0 | 43 |
|  | 0048 | 1/0.02 | 7/0.2 | 1/0.4 | 0/0 | 0/0 | 0/0 | 0/0 | 3/0.6 | 7/2 | 19 |
|  | 0224 | 27/0.4 | 13/0.3 | 0/0 | 3/0.4 | 2/0.4 | 1/0.2 | 6/1.0 | 1/0.2 | 1/0.3 | 54 |
|  | 1123 | 16/0.3 | 1/0.02 | 0/0 | 0/0 | 1/0.2 | 0/0 | 0/0 | 0/0 | 1/0.3 | 19 |
|  | 0073 | 3/0.05 | 0/0 | 0/0 | 0/0 | 0/0 | 0/0 | 0/0 | 1/0.2 | 1/0.3 | 5 |
|  | **Ave.** | 16/0.3 | 5.6/0.1 | 0.3/0.1 | 1/0.1 | 0.8/0.2 | 1.1/0.2 | 1.6/0.3 | 1.4/0.3 | 2.1/0.6 | 29.9 |
| Nonsynonymous | 0096 | 15/0.2 | 7/0.2 | 0/0 | 5/0.7 | 9/1.6 | 4/0.9 | 3/0.5 | 0/0 | 1/0.3 | 44 |
|  | 0045 | 25/0.4 | 1/0.02 | 0/0 | 1/0.1 | 0/0 | 0/0 | 2/0.3 | 0/0 | 0/0 | 29 |
|  | 0046 | 26/0.4 | 2/0.05 | 2/0.9 | 2/0.3 | 0/0 | 0/0 | 1/0.2 | 5/1.0 | 4/1.1 | 42 |
|  | 0047 | 91/1.5 | 8/0.2 | 3/1.3 | 4/0.5 | 0/0 | 4/0.9 | 20/3.2 | 8/1.6 | 0/0 | 138 |
|  | 0048 | 9/0.1 | 8/0.2 | 0/0 | 0/0 | 1/0.2 | 0/0 | 4/0.6 | 11/2.1 | 17/4.9 | 50 |
|  | 0224 | 23/0.4 | 6/0.1 | 1/0.4 | 3/0.4 | 6/1.1 | 1/0.2 | 6/1.0 | 2/0.4 | 4/1.1 | 52 |
|  | 1123 | 28/0.5 | 5/0.1 | 0/0 | 0/0 | 1/0.2 | 1/0.2 | 1/0.2 | 2/0.4 | 0/0 | 38 |
|  | 0073 | 5/0.08 | 1/0.02 | 0/0 | 0/0 | 0/0 | 0/0 | 0/0 | 7/1.4 | 4/1.1 | 17 |
|  | **Ave.** | 27.8/0.4 | 4.8/0.1 | 0.5/0.3 | 1.9/0.3 | 2.1/0.4 | 1.3/0.3 | 4.6/0.7 | 4.3/0.9 | 3.8/1.1 | 51.3 |
| Frame shift,  No-stop,  Nonsense,  In-frame insertion or deletion | 0096 | 1/0.02 | 0/0 | 0/0 | 0/0 | 0/0 | 0/0 | 0/0 | 1/0.2 | 0/0 | 2 |
|  | 0045 | 7/0.1 | 0/0 | 1/0.4 | 0/0 | 0/0 | 0/0 | 0/0 | 0/0 | 2/0.6 | 10 |
|  | 0046 | 1/0.02 | 0/0 | 0/0 | 0/0 | 0/0 | 0/0 | 1/0.2 | 1/0.2 | 1/0.3 | 4 |
|  | 0047 | 7/0.1 | 0/0 | 0/0 | 0/0 | 0/0 | 0/0 | 6/1.0 | 4/0.8 | 0/0 | 17 |
|  | 0048 | 0/0 | 1/0.02 | 0/0 | 0/0 | 0/0 | 0/0 | 0/0 | 0/0 | 3/0.9 | 4 |
|  | 0224 | 1/0.02 | 2/0.05 | 0/0 | 0/0 | 1/0.2 | 0/0 | 0/0 | 0/0 | 0/0 | 4 |
|  | 1123 | 1/0.02 | 0/0 | 0/0 | 0/0 | 0/0 | 0/0 | 0/0 | 0/0 | 0/0 | 1 |
|  | 0073 | 0/0 | 0/0 | 0/0 | 0/0 | 1/0.2 | 1/0.2 | 0/0 | 0/0 | 0/0 | 2 |
|  | Total | 2.3/0.04 | 0.4/0.01 | 0.1/0.05 | 0/0 | 0.3/0.05 | 0.1/0.03 | 0.9/0.15 | 0.8/0.15 | 0.8/0.2 | 5.5 |

^a^; ORF SNPs for each SNP category and virus strain.

^b^; percentage of SNPs
